# A Bayesian Time-Varying Psychophysiological Interaction Model

**DOI:** 10.1080/26941899.2025.2519436

**Published:** 2025-06-24

**Authors:** Brian Schetzsle, Jaylen Lee, Aaron Bornstein, Babak Shahbaba, Michele Guindani

**Affiliations:** a Department of Statistics, University of California, Irvine, California, USA; b Department of Cognitive Sciences, University of California, Irvine, California, USA; c Department of Biostatistics, University of California, Los Angeles, California, USA

**Keywords:** Psychophysiological interaction, time-varying parameter, dynamic covariance, functional connectivity

## Abstract

Functional connectivity, the study of coordination between distinct brain regions, is a key focus in neuroscience. The Psychophysiological Interaction (PPI) model, commonly used to infer task-dependent functional connectivity, is limited by its susceptibility to confounding effects. We propose using partial correlations, instead of PPI regression coefficients, as they correct for confounding. We show how the PPI model can be used to estimate the precision matrix of a Gaussian Graphical Model (GGM), from which partial correlations are easily derived. We then propose a Bayesian extension to the PPI model that allows this measure of functional connectivity to vary over time. We enforce sparsity in the GGM precision matrix through scale-mixture shrinkage priors, mitigating overfitting. Additionally, we identify structural zeros in the precision matrix using a Bayesian multicomparison decision-theoretic framework. We demonstrate the efficacy of our model over the standard PPI model using simulated data and we further apply it to human fMRI data from a serial reaction time experiment. Our framework offers a more robust and dynamic approach to functional connectivity analysis.

## 1. Introduction

The study of coordination of activity between brain regions, known as functional connectivity (FC), has led to a better understanding of brain mechanisms, including how the brain responds to external stimuli. Those advances have also resulted in improved diagnoses and classifications of disease severity (see, e.g. Lynall et al. (2010); Anderson and Cohen (2013)). Friston (2011) defines FC as the study of the (undirected) statistical dependencies between brain regions.

The psychophysiological interaction (PPI) model was first proposed by Friston et al. (1997) as a formal way of investigating/quantifying these statistical dependencies. This model is typically applied in task-based fMRI studies wherein the subject is exposed to an experimental stimulus in the form of a task while undergoing repeated MRI scans. The goal is to identify the effect this stimulus has on the coordinated activity between brain regions as measured by the blood-oxygen level dependent (BOLD) response recorded by the MRI scans. The PPI model makes the reasonable assumption that two brain regions exhibit coordinated activity if one region’s activity is predictive of the other. This is formalized as a linear regression with one brain region’s BOLD response as the dependent variable and other regions of interest (ROIs) as predictors. The modulatory effect of the experimental stimulus is captured by the model through the inclusion of interaction terms. The original model by Friston et al. (1997), which considered only a single stimulus, was later generalized by McLaren et al. (2012) to accommodate multiple stimuli.This generalized PPI model (gPPI) has become so ubiquitous that when the PPI model is invoked it is often referring to its generalized form.

While the main contribution of this paper is a time-varying extension of the PPI model, we first bring attention to an appealing interpretation of the coefficients in the standard PPI model. We note how the PPI model for modeling one region’s activity as a function of the other regions is consistent with a Gaussian Graphical Model for brain activity as a whole. Partial correlation, which is the correlation unique to a pair of variables conditional on all the others, is a quantity that arises easily from a Gaussian Graphical Model.

The PPI model is widely purported to be vulnerable to mediating effects of other brain regions (see, for instance, Gitelman et al. (2003)). The implication is that the coefficients can only be used to identify the presence of coordination without any sense of its magnitude. Using partial correlation between brain regions as a measure of functional connectivity overcomes the potential limitations of mediating effects and makes the magnitude of functional connectivity directly interpretable. We note how the values of the coefficients in a saturated PPI model, i.e. one that includes all brain regions, are proportional to the corresponding partial correlations. Moreover, deriving the partial correlation between each pair of brain regions is computationally straightforward.

Recent developments in neuroimaging suggest that inter-regional coordination in the absence of stimuli may better be characterized as varying over time (Calhoun et al. 2014; Chen and Leng 2016; Iraji et al. 2019). This flexibility is not supported by the generalized PPI model of McLaren et al. (2012) and may obscure the modulatory effects of experimental stimuli. Our main contribution in this paper is a further generalization of the PPI model that can accommodate potentially time-varying connectivity. We do this using a Bayesian framework which allows us to model partial correlations as dynamic processes. Placing continuous scale-mixture shrinkage priors on the means and variances of these processes simultaneously avoids overfitting and encourages the processes to “shrink” to a time-invariant values in the absence of evidence to the contrary.

Our second contribution is the development of a new method for identifying significant inter-regional partial correlations by adapting a non-marginal decision-theory-based multicomparison framework proposed by Chandra and Bhattacharya (2019). This multiple testing procedure is designed to control the rate of false-positive decisions (concluding that two brain regions’ activity are coordinated when they are not) while still allowing the model to detect true non-zero partial correlations.

The paper is structured as follows. In Section 2.1, we define the PPI model and explain its link with the Gaussian Graphical Model. In Section 2.2 we introduce our Bayesian Time-varying PPI model and describe the shrinkage priors applied to the model coefficients. Section 2.3 describes how we obtain posterior inference of partial correlations from our model as well as details on the multicomparison procedure for testing the significance of those partial correlations. We illustrate the performance of our model in a simulation setting (Section 3.2) and in an application to data from a serial reaction time experiment (Section 3.4). Finally, Section 4 concludes with a discussion of our proposed method, as well as plans for future investigation.

## 2. Methods

### 2.1. The PPI Model

In this section we review the popular PPI model of Friston (2011) and its generalization proposed by McLaren et al. (2012). We also review the Gaussian Graphical Model, which can be employed as a working model for describing brain connectivity. We show that the PPI model estimates the mean and inverse covariance of a Gaussian Graphical Model and that the fitted coefficients of the PPI model can be used to derive partial correlations between brain regions.

The PPI model originally proposed by Friston seeks to quantify the coordination of activity between functionally distinct brain regions, especially in response to an experimental stimulus. Its validity rests on the reasonable assumption that two regions are functionally connected if activity in one region is predictive of activity in the other. In this section, we restate the form of this model, with some minor adjustments and their justifications to build the framework for the extensions presented in the next Section.

In fMRI studies, the MRI machine records changes in the amount of oxygenated hemoglobin in small volumetric pixels, voxels, of the brain at consistent time intervals over the course of the study. Oxygenated blood flows into regions of the brain that have recently experienced neural activity, synaptic firing, in order to “reset” the synapses. The blood oxygen level dependent (BOLD) response recorded by the MRI machine thus serves as a proxy for neural activity. However, that response does not happen instantaneously but instead oxygenated hemoglobin rushes into a region that has experienced neural activity, peaking after about 6 s and is then gradually flushed from the area. The BOLD response observed in a voxel Y(t) is frequently modeled as the convolution of an underlying neural signal $y_{j}(t)$ with a hemodynamic response function (HRF) which we define as h(t). In the following, we denote this convolution as Y(t)=H(y(t)) where the function H(⋅) convolves its operand with the HRF h(t), i.e. H(y(t))=⟨h(t)*y(t)⟩. Through a process of deconvolution the implicit neural activity y(t) can be recovered. We denote this deconvolution as $y(t)=H^{-1}(Y(t))$. Gitelman et al. (2003) suggest several ways to perform this deconvolution and propose a Bayesian method that we will discuss later.

When using the PPI model for functional connectivity analysis, the predictor and response regions are typically chosen ahead of time, often based on available prior literature or a testable research hypothesis. The predictor regions are referred to as *seed* regions and the response region as the *target*. Seed-based functional connectivity analysis investigates the association in the fMRI signal of each seed region to the target region. This approach is fundamentally bivariate in nature, as the associations are found independently for each seed-target pair. However, the results are heavily dependent on the choice of seed and target regions; if different seed regions are chosen the results may vary. Thus, the choice of seed regions can impact the reproducibility and generalizability of any findings (Lv et al. 2018). A possible solution is to include all regions in the model, one as the target and the others as seeds. Traditional methods for testing for significant associations among many regressors, such as p-values which are typically used in PPI analysis, are known to detect spurious non-zero effects. In Section 2.3.1 we propose a method that is more robust against this type of error. First we explore the implications of including all regions in the PPI model.

In an fMRI study, the voxels are typically aggregated into P functionally distinct regions of the brain, and activity within each of the constituent voxels is aggregated so that $Y_{i}(t),i=1,\ldots ,P$ represents the average BOLD response of the voxels in the region i. A study will also have K experimental conditions or stimuli. Let $s_{k}(t)$ denote the strength of stimulus k at time t. In an experiment with a block design or impulse design $s_{k}(t)=1$ when the experimental stimulus is active and $s_{k}(t)=0$ otherwise. In a more general scenario, the experimental stimulus could be thought also as varying continuously over time. We will explore this situation further, both in our simulation and in our application.

Now, given aggregated BOLD response data $Y_{i}(t)$ and their implied neural-level activity $y_{i}(t),i=1,\ldots ,P$ as well as the K experimental stimuli $s_{k}(t),k=1,\ldots ,K$, we consider the generalized form of the PPI model:

(1) $$Y_{i}(t)=\mu_{i}\;\;+\underset{\text{physiological}}{\underbrace{\sum_{j\neq i}\;\beta_{i}^{j}Y_{j}(t)}}+\underset{\text{psychological}}{\underbrace{\sum_{k=1}^{K}\;\alpha_{i,k}H\left(s_{k}(t)\right)}}+\underset{\text{psychophysiological}\;\text{interaction}}{\underbrace{\sum_{k=1}^{K}\;\sum_{j\neq i}\;\gamma_{i,k}^{j}H\left(s_{k}(t)y_{j}(t)\right)}}+\epsilon_{i}(t)$$

We should note that this formulation differs in a few respects from the generalized PPI model of McLaren et al. (2012). For instance, we include a mean term $\mu_{i}$ because we don’t wish to assume our data has been de-meaned prior to analysis. We do, however, assume that the data has had all confounding effects (e.g. subject movement within the MRI machine) removed, a standard procedure prior to analysis. The model residuals $e_{i}(t)$ we assume to be normally distributed, centered at 0. We also incorporate the work of Gitelman et al. (2003), who stress the distinction between neural-level and hemodynamic-level data, by calculating the psychophysiological interaction regressor at the neural-level rather than the hemodynamic-level before convolving that product with the HRF. This is represented in the term $H\left(s_{k}(t)y_{j}(t)\right)$ in the psychophysiological interaction portion of equation (1) and which we clarify below.

There is no generally accepted method for convolving and deconvolving an HRF. The method proposed by Gitelman et al. (2003) infers likely neuronal activity $y_{i}(t)$ from observed BOLD response $Y_{i}(t)$ using Bayesian priors on the weights of a set of user-specified basis functions. This method is used in the popular fMRI analysis software package SPM. Regularization inherent in the estimation of the weights results in imperfect reconvolution of a deconvolved time-series. We were uncomfortable with this so instead employ a more simplified method of deconvolution that relies on time-shifted canonical HRF basis functions and least squares estimates of weights. This method yields interaction regressors that factor as $S(t)Y_{i}(t)$, where S(t) is the stimulus s(t) convolved with the canonical HRF, i.e. $H\left(s_{k}(t)y_{i}(t)\right)=H\left(s_{k}(t)\right)Y_{i}(t)$ (Wu et al. 2013). By defining $S_{k}(t)=H\left(s_{k}(t)\right)$, i.e. stimulus k convolved with the HRF, then we can re-express equation (1) as

(2) $$Y_{i}(t)=\left(\mu_{i}+\sum_{k=1}^{K}\;\alpha_{i,k}S_{k}(t)\right)+\sum_{j\neq i}\;\left(\beta_{i}^{j}+\sum_{k=1}^{K}\;\gamma_{i,k}^{j}S_{k}(t)\right)\;\;Y_{j}(t)+\epsilon_{i}(t)$$

This arrangement allows $Y_{i}(t)$ to be factored out of $H\left(s_{k}(t)y_{i}(t)\right)$. Our choice of basis functions in the deconvolution algorithm allow this directly but we point out that this factorization is still possible with other selections of basis functions or other methods of deconvolution, by defining a new stimulus vector at the hemodynamic-level, $S_{k}^{*}(t)=\frac{H\left(s_{k}(t)y_{i}(t)\right)}{Y_{i}(t)}$. This is properly defined as long as $Y_{i}(t)\neq 0$, which is one of our motivations for not de-meaning the data.

Furthermore, equation (2) clearly shows that the arrangement of the PPI model is an example of a varying-coefficient model (Cleveland et al. 1992; Hastie and Tibshirani 1993). This class of models is linear in the regressors but the coefficients are allowed to change smoothly as a function of another set of variables, which can be seen as “effect modifiers.” In the PPI literature the experimental stimuli are indeed often considered as effect modifiers; however, as we will discuss later, effect modifiers do not need to be related only to the stimulus effect.

More importantly, equation (2) can be related to Gaussian Graphical Models (GGMs). A GGM provides an appealing working model for studying whole brain activity and inter-regional coordination. The nodes represent functionally distinct regions of the brain and an edge connecting two nodes represents an undirected relationship between those two nodes. A GGM is defined through a multivariate normal distribution characterized by a mean vector μ and a covariance matrix Σ. The inverse of Σ, i.e. the precision matrix $\Omega =\Sigma^{-1}$, encodes conditional independences between the nodes, i.e. the connections in the graph (Dempster 1972; Lauritzen 1996). Two nodes are connected by an edge if their partial correlation is different than zero. In this context, partial correlation captures the correlation between activity in two brain regions conditional on all other regions’ actvity, i.e. the correlation that cannot be explained by any other region. In formulas,

(3) $$\rho_{Y_{i},Y_{j}|Y/\left\{Y_{i},Y_{j}\right\}}=-\frac{\Omega_{i,j}}{\sqrt{\Omega_{i,i}\Omega_{j,j}}},$$

i,j=1,…,P. Partial correlation is an appealing measure of functional connectivity because it resolves the problem of mediating effects, a well-known limitation of the PPI model (Gitelman et al. 2003).

We can reformulate the PPI model (2) as a GGM as follows. Let $Y(t)∼{𝒩}_{P}(\mu (t),\Sigma (t))$ be a P-dimensional multivariate Gaussian random vector where $Y=\left[Y_{1}(t),\ldots \right.\;,\;{\left.Y_{P}(t)\right]}^{T},\mu (t)=\left[\mu_{1}(t),\ldots ,\mu_{P}(t)\right]$ and $\Sigma (t)=\left[\Sigma_{ij}(t)\right],\;i,j=1,\ldots ,P$. Let $\Omega (t)=\Sigma^{-1}(t)$. Then, it is possible to represent a GGM through a series of regressions by conditioning each node $Y_{i}$ on all other nodes $Y_{-i}$ (Maathuis et al. 2018; Tsai et al. 2022). More specifically, we can write

(4) $$Y_{i}(t)=\mu_{i}^{*}(t)+\sum_{i\neq j}B_{i}^{j}(t)Y_{j}(t)+\epsilon_{i}(t)$$

where $B_{i}^{j}(t)=\beta_{i}^{j}+\sum_{k=1}^{K}\gamma_{i,k}^{j}S_{k}(t)$ and $\mu_{i}^{*}(t)=\mu_{i}+\sum_{k=1}^{K}\alpha_{i,k}S_{k}(t)$ Thus, the regression coefficients $B_{i}^{j}(t)$ consist of two components: one that quantifies the background partial correlation and another that represents a change in the partial correlation induced by experimental stimuli. Moreover, we can obtain an expression of time-varying partial correlations,

(5) $$\rho_{Y_{i},Y_{j}|Y/\left\{Y_{i},Y_{j}\right\}}(t)=\left(-\sqrt{\frac{\Omega_{i,i}(t)}{\Omega_{j,j}(t)}}\right)B_{i}^{j}(t),$$

i.e. the coefficients $B_{i}^{j}(t)$ in (4) are proportional to the partial correlations between nodes of a GGM. Thus, if a Gaussian Graphical Model is adopted as the working model for whole-brain activity, then the standard gPPI model can be used to estimate the rows of the precision matrix at time t,Ω(t), which can, in turn, be used to derive partial correlations between regions.

Meinshausen and Bühlmann (2006) called the row-wise estimation of a precision matrix “neighborhood selection” because each node in a graph corresponds to a row of a precision matrix, and the non-zero entries in that row correspond to the node’s neighbors. They proposed estimating the rows of a time-invariant precision matrix using the penalized regression Lasso (Tibshirani 1996). Recent work attempting to estimate a dynamic covariance or precision matrix that changes over time uses kernel smoothing (Chen and Leng 2016) or penalized basis splines (Xue et al. 2020). Our proposed method allows for flexibly modeling a precision matrix that varies both over time and in response to an external stimulus.

### 2.2. A Bayesian Time-Varying PPI Model

In this section, we present our proposed Bayesian Time-varying Psychophysiological Interaction (PPI) model, which extends the standard gPPI model by allowing all regression coefficients to vary over time. This extension accounts for the fact that time-dependence is not solely induced by experimental stimuli but also by the non-stationary nature of resting-state (background) functional connectivity, thus providing a more realistic representation of the underlying neural processes. More precisely, dynamics in $B_{i}^{j}(t)$ can be achieved by allowing the coefficients $\beta_{i}^{j}$ and $\gamma_{i,k}^{j}$ to vary. Additionally, $\mu_{i}$ and $\alpha_{i,k}$ can also be allowed to vary to increase the flexibility in the intercept term $\mu_{i}^{*}$, preventing larger variance in the residuals $\epsilon_{i}$ and, consequently, poor estimates of the diagonal elements of the precision matrix $\Omega_{i,i}(t)$ from which they are derived. Our model, then, takes the form

(6) $$\begin{array}{l} Y_{i}(t)=\left(\mu_{i}(t)+\sum_{k=1}^{K}\;\alpha_{i,k}(t)S_{k}(t)\right)+\\ \;\sum_{j\neq i}\;\left(\beta_{i}^{j}(t)+\sum_{k=1}^{K}\;\gamma_{i,k}^{j}(t)S_{k}(t)\right)Y_{j}(t)+\epsilon_{i}(t). \end{array}$$

**Figure 1.**
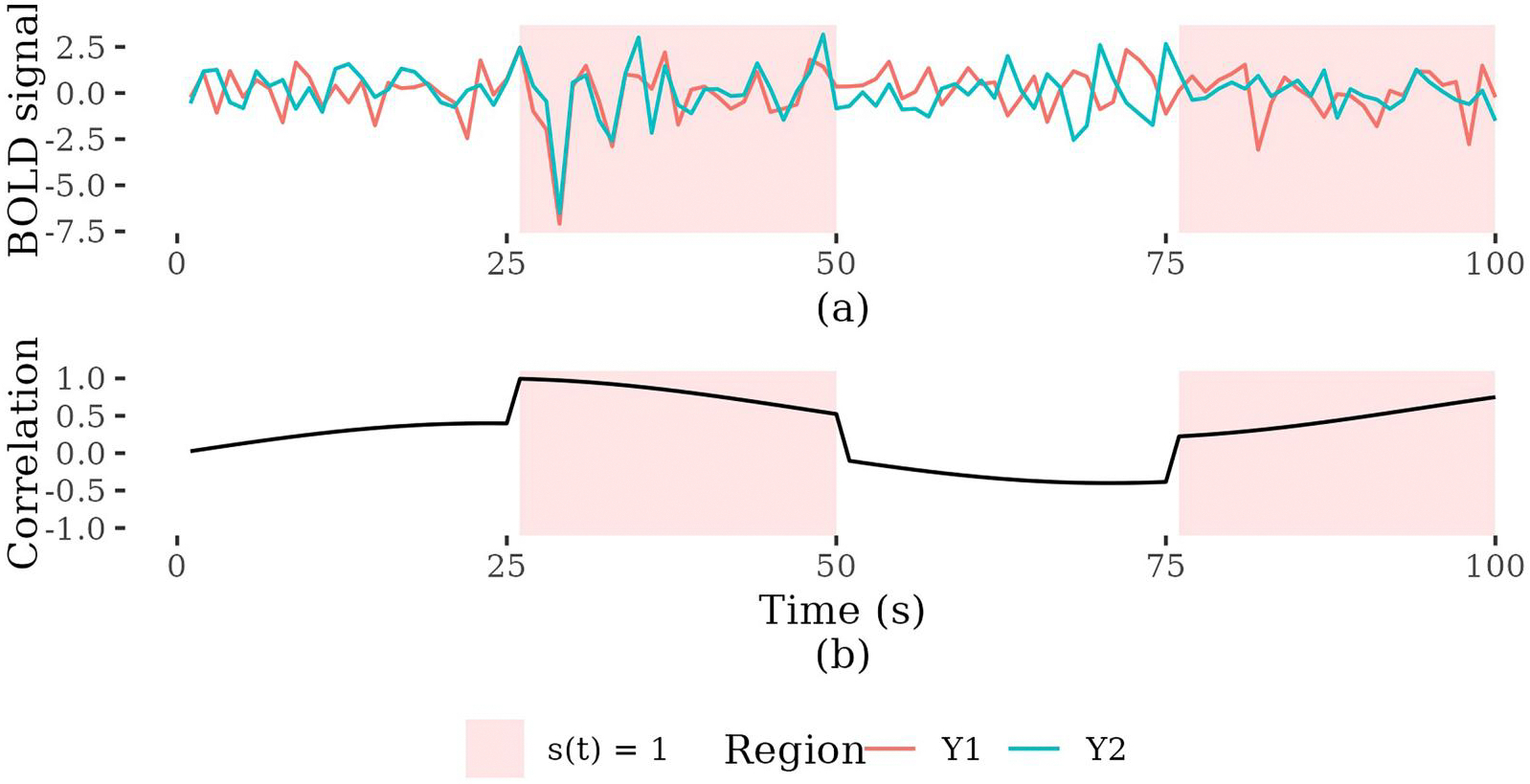
This simulated data set illustrates the two dynamics that our proposed model is able to capture. Here, background connectivity as measured by partial correlation varies smoothly over time. There is an additional change in partial correlation when the experimental stimulus is active, when s(t)=1. By comparison, the Generalized PPI is only able to model the stimulus-dependent changes in partial correlation.

Figure 1 provides an illustrative example of the type of time-varying relationship between two time series that our proposed model can capture. We generate bi-variate Gaussian data with a correlation structure that varies both smoothly over time and abruptly in response to a stimulus. We obtain the partial correlation between the two variables as $\rho_{1,2}(t)=0.4\cdot sin(t/15)+0.6\cdot s(t)$, where the stimulus process s(t) alternates between periods of activity and inactivity every 25 s.

The two simulated time series in Figure 1 are only weakly dependent when s(t)=0. That dependence increases when s(t)=1, which manifests as greater concordance between the two time-series during periods where s(t)=1. The time-dynamic changes in partial correlation are modeled by β(t) while the stimulus-dependent changes in partial correlation come from γ(t). The existing non-dynamic versions of the PPI model are not capable of capturing these subtle fluctuations in the strength of inter-regional connectivity.

Friston et al. (1997) emphasizes that, in general, PPI coefficients should not be interpreted as correlations, though testing their significance is equivalent to testing for the significance of correlations. A key advantage of our model, which includes all regions, is that the coefficients *can* be interpreted as partial correlations between regions, as discussed in Section 2.2. This makes the magnitude of the fitted coefficients meaningful. Partial correlations are particularly advantageous because they account for mediating effects of other regions, a frequently cited limitation of the PPI model. To reiterate, the standard PPI coefficients are used as a tool to test for the presence of functional connectivity between regions; beyond that their magnitude defies interpretation. The magnitude of the PPI coefficients in our proposed model are directly interpretable as partial correlations. They give a sense of the strength of functional connectivity between brain regions while controlling for mediating effects of other regions.

Building on the derivation of partial correlations in the non-dynamic PPI model of Section 2, the partial correlation between a seed region j and a target region i can be derived from (6) as

(7) $$\rho_{i,j|}(t)=\left(\beta_{j}^{i}(t)+\sum_{k=1}^{K}\;S_{k}(t)\cdot \gamma_{k,j}^{i}\right)\frac{\sigma_{j}}{\sigma_{i}},\;i\neq j.$$

Here, $\beta_{j}^{i}(t),\gamma_{j,k}^{i}$, and $\sigma_{i}$ are obtained from the PPI model where region i is the target, while $\sigma_{j}$ is derived from a separate PPI model where region j is the target. Thus, calculating any partial correlation requires fitting two PPI models. Fortunately, fitting the P PPI models, one for each target region, yields all the estimates of the parameters necessary to calculate the partial correlation between any two regions. These P regressions can be done efficiently in parallel. However, there is no guarantee that estimates of $\rho_{i,j}(t)$ and $\rho_{j,i}(t)$ align. In fact, one may be deemed significant while the other may not, which presents an evident problem. Asymmetry in the precision matrix would imply directionality of association which is assumed not to be the case in functional connectivity analysis. Furthermore, an asymmetric precision matrix is incompatible with a Gaussian Graphical Model, our working model for whole brain activity. In Section 2.3.1 we describe a multi-comparison procedure to detect structural zeros and enforce symmetry in the precision matrix.

#### 2.2.1. Shrinkage Priors

The time-varying coefficients capture varying connectivity between the seed region j and the target region i. At any given time t, it is reasonable to expect that only a subset of the seed regions will be related to the target, i.e. we expect a degree of sparsity among the associations. Our Bayesian treatment of the PPI model gives us needed flexibility in specifying the behavior of our model’s coefficients by placing appropriate sparsity-inducing prior distributions on them. Recently, several Bayesian approaches have been proposed for dynamic variable selection priors in sparse time-varying state-space models (see for example Kowal et al. 2019; Rockova and McAlinn 2020). Here, we employ the double gamma prior, which was recently proposed by Bitto and Frühwirth-Schnatter (2019) and Cadonna et al. (2020). This prior generalizes the popular Horseshoe prior (Carvalho et al. 2010) to the time-varying modeling framework. In our experience, this choice of prior is efficient from a computational perspective, helps to avoid overfitting by restricting the variances of the time-varying coefficients, and is flexible enough to adapt to varying degrees of sparsity. We give details of the double-gamma formulation in this section.

We model all the regression coefficients in equation (6) dynamically in the same way. To simplify the notation, we re-express the model by defining the vector $\eta_{i}(t)=\left[\mu_{i}(t),\alpha_{i}(t),\beta_{i}(t),\gamma_{i}(t)\right]$, which includes all P(K+1) regression coefficients. Let $\eta_{i}^{j}(t)$ be the j-th component of the vector $\eta_{i}(t)$. We assume that these regression coefficients exhibit a relatively smooth temporal evolution. A class of processes flexible enough to capture a variety of possible time courses is the discrete random walk. Thus, we consider

(8) $$\eta_{i}^{j}\left(t\right)=\eta_{i}^{j}\left(t-1\right)+\omega_{i}^{j}\left(t\right),$$

where the $\omega_{i}^{j}(t),t=1,\ldots ,N$, are independent identically distributed random variables centered at 0. The degree of variability in the path of $\eta_{i}^{j}(t)$ over time is driven by the variability of $\omega_{i}^{j}(t)$; if the variance of $\omega_{i}^{j}(t)$ shrinks to 0, there are no changes to $\eta_{i}^{j}(t)$ over time and it degenerates to a constant value, that being its starting point $\eta_{i}^{j}(0)$. The double-gamma prior of Bitto and Frühwirth-Schnatter (2019) places a normal prior on $\omega_{i}^{j}(t)$ and controls the variance with two gamma distributions:

(9) $$\eta_{i}^{j}(t)=\eta_{i}^{j}(t-1)+\omega_{i}^{j}(t)$$

$$\omega_{i}^{j}(t)|\theta_{i}^{j}\overset{iid}{∼}𝒩\left(0,\theta_{i}^{j}\right)\;t=1,\ldots ,N$$

$$\theta_{i}^{j}|\;\xi_{i}^{j_{2}}∼𝒢\left(\frac{1}{2},\frac{1}{2\xi_{i}^{j_{2}}}\right)$$

$$\xi_{i}^{j_{2}}|a^{\xi },\kappa^{2}\overset{iid}{∼}𝒢\left(a^{\xi },\frac{a^{\xi }\kappa^{2}}{2}\right)\;j=1,\ldots ,P(K+1)$$

Restricting the variance of a random walk process when it is used as a time-varying parameter in a model is necessary to avoid overfitting. We are simultaneously interested in maintaining sparsity in our model. We achieve this by also placing restrictions on the mean of the random walk process. Thus, a similar double-gamma shrinkage prior formulation is placed also on the starting value of the process $\eta_{i}^{j}(0)$, to encourage the process to be centered at 0 in the absence of evidence from the data to the contrary. If the variance of the random walk process also shrinks to 0, then the parameter degenerates further to a constant 0 and effectively drops out of the model. Appealingly, this situation is not difficult to achieve because those values are interior points in the support of $\eta_{i}^{j}(t)$ and $\theta_{i}^{j}$. If $\theta_{i}^{j}=0$ but $\eta_{i}^{j}(0)\neq 0$ then the corresponding functional connectivity component is present but not dynamic. If $\theta_{i}^{j}\neq 0$ and $\eta_{i}^{j}(0)\neq 0$ then the component is present and dynamic.

The double-gamma prior in Equation (9) is an example of a *global-local* shrinkage prior (Polson and Scott 2012). The parameter κ is the global shrinkage parameter common to all $\theta_{i}^{j}$. Larger values of this parameter encourage all values of $\theta_{i}^{j}$ to shrink toward 0. The parameter $a^{\xi }$ controls local adaptation to the global level of shrinkage induced by κ. Specifically, larger values of $a^{\xi }$ enable individual $\theta_{j}$ to better counterbalance the global shrinkage effect, allowing for more flexibility. Bitto and Frühwirth-Schnatter (2019) have excellent illustrations of how different values of the parameters $a^{\xi }$ and κ affect the shrinkage of process variances to 0 in the time-varying parameter model. The choice of these parameters should ideally be informed by prior knowledge regarding the expected variability in components of functional connectivity. Alternatively, these parameters can be learned from the data, which is the approach we have adopted.

### 2.3. Posterior Inference of Model Coefficients

In this section, we outline how we obtain inference on the coefficients of our model. Since the posterior distribution is not available in closed form, we need to use posterior samples obtained *via* a Markov Chain Monte Carlo (MCMC) algorithm for posterior inference. Fortunately, the model in (6) can be simply implemented in the R package *shrinkTVP* by Knaus et al. (2021), which uses the collapsed sampler employed by Cadonna et al. (2020) to efficiently perform MCMC sampling of the coefficients in a regression model with double-gamma priors on the regression coefficients. This package also enables inference on stochastic volatility, allowing our model to capture structured changes in the error variance of equation (6). Since the diagonal elements of Ω(t), crucial for calculating partial correlations, are the inverse of the error variance, accurately modeling the error variance is essential. Our BTV-PPI model treats region i as the target and all other regions as seeds to model the elements of the ith row of Ω(t). We fit P separate and independent BTV-PPI models to estimate all rows of Ω(t). Each row is fit independently so we can do this efficiently in parallel, leading to a sizeable computational gain. Next, we provide a decision-theoretic framework for testing the significance of the resulting partial correlations and ensuring the symmetry of a final estimate of Ω(t), inspired by the work of Chandra and Bhattacharya (2019). We then discuss the procedure for constructing point estimates of the time-varying partial correlation matrices, $G_{t}\in {ℛ}^{P\times P}\;t=1,\ldots ,T$.

#### 2.3.1. Graph Selection

After performing the MCMC algorithm for each of the P rows of Ω(t), we obtain posterior samples for each cell of the dynamic precision matrix Ω(t). In other words we get samples of $\Omega_{ij}(t)$. We arrange samples of these individual cells to form a single unified sample of the dynamic precision matrix Ω(t). For instance, by arranging the first MCMC sample of $\Omega_{ij}(t)\;\forall i,j$ in a 3D matrix we form the first MCMC sample of Ω(t). However, this estimated Ω(t) has several shortcomings that need to be addressed. First, the estimated Ω(t) does not reflect the assumed sparsity of connections between different brain regions. This is due to the nature of continuous shrinkage priors, which induce shrinkage toward 0 without discontinuities at 0. In contrast, discrete spike-and-slab priors (George and McCulloch 1993, 1997) can place positive posterior probability mass on zero, allowing for easy computation of the posterior probability of inclusion of coefficients in the model. However, their use comes at a significant computational cost. Since continuous shrinkage priors never take the exact value 0, it is necessary to establish criteria for determining when a coefficient is statistically different from 0. Thresholding approaches have been proposed, where a posterior sample value is set to exactly 0 if its value is within a small range around zero. Alternative techniques, such as those suggested by Carvalho et al. (2010) and Cadonna et al. (2020), set a threshold based on a latent shrinkage factor of a coefficient. Such thresholding methods do not formally account for multiple comparisons, as we need for testing the significance of P×(P-1) off-diagonal elements at each time point t=1,…,N. Another important limitation of the estimated Ω(t) is that it is not symmetric. This arises from the rows of Ω(t) being fit independently, as described below. Lack of symmetry is a problem because the resulting estimate cannot be a valid precision matrix. In the following, we will explain how we correct for the lack of symmetry *and* account for multiple comparisons to address these limitations of the estimated Ω(t).

The partial correlation between two brain regions is proportional to the off-diagonal element of the precision matrix $\Omega_{ij}(t)$. Due to our row-wise estimation of Ω(t), our approach leads to two estimates of this element: $\Omega_{ij}(t)$ from the fitted BTV-PPI model with region i as the target, and $\Omega_{ji}(t)$ from the model with region j as the target. Conducting a marginal test that considers $\Omega_{ij}(t)$ and $\Omega_{ji}(t)$ in isolation can lead to conflicting conclusions. This is because the two estimates are not independent, as they arise from the same underlying connectivity pattern between regions i and j. Testing each estimate separately may result in one estimate being deemed significant while the other is not, even though they represent the same partial correlation.

Chandra and Bhattacharya (2019) developed a Bayesian non-marginal decision-theoretic approach to address hypothesis testing with dependent hypotheses, emphasizing concordant conclusions among sets of related hypotheses. Here, non-marginal means that the joint posterior distribution of $\Omega_{ij}(t)$ and $\Omega_{ji}(t)$ is considered, rather than relying solely on marginal decision rules based on the marginal posterior distribution of $\Omega_{ij}(t)$ alone. This approach leads to a modified false discovery rate (mFDR) criterion that is more accurate than marginal methods.

To define the set of dependent hypotheses, let $H_{0,i,j,t}\;:\;\Omega_{i,j}(t)=0$ be the hypothesis that the off-diagonal element $\Omega_{ij}(t)$ is 0 with the corresponding alternative hypothesis $H_{A,i,j,t}:\Omega_{i,j}(t)\neq 0$, indicating that it is not 0. The set of related hypotheses is then defined as ${ℋ}_{i,j,t}=\left\{H_{0,i,j,t}\right.,\left.H_{A,i,j,t},H_{0,j,i,t},H_{A,j,i,t}\right\}$. Let $D_{i,j,t}=I\;(H_{A,i,j,t}$ is accepted) be our decision function, which takes the value 1 when we conclude significance of $\Omega_{i,j}(t)$. For simplicity of notation, it is convenient to introduce a variable $z_{i,j,t}=D_{j,i,t}$, which simply allows us to state the related decision about the significance of $\Omega_{j,i}(t)$ using the same index. Lastly, let $h_{i,j,t}=I\left(\Omega_{i,j}(t)\neq 0\right)$ indicate the true state of nature, taking the value of 1 if regions i and j are truly functionally connected. Let ${𝒜}_{t}$ denote the set of triplets (i,j,t) formed as we vary i,j=1,…,R,i≠j, at each time t=1,…,T. Following Chandra and Bhattacharya (2019), a non-marginal decision rule will aim at maximizing the posterior expectation of true positive (TP) decisions at each time t,

(10) $$TP_{t}=\sum_{i\in {𝒜}_{t}}D_{i}h_{i}z_{i}$$

while controlling for possible decision errors. $TP_{t}$ counts the number of cases where both $H_{A,i,j,t}$ and $H_{A,j,i,t}$ are correctly accepted.

In the context of non-marginal decision rules there are several sources of decision errors: related hypotheses are both wrongly accepted, both wrongly rejected, or do not agree. Chandra and Bhattacharya (2019) show that a count of these errors adds up to the following total error,

(11) $$E_{t}=\sum_{i\in {𝒜}_{t}}D_{i}\left(1-h_{i}z_{i}\right).$$

The resulting optimization problem can be seen as a constrained minimization of a loss function that penalizes both false positive decisions and conflicting decisions while maximizing the posterior expectation of true positive decisions. Thus, a general objective function can be written as a function of the admissible decisions in the set of all possible decision configurations under some penalization constant,

(12) $$f_{\eta ,t}(D)=\sum_{i\in {𝒜}_{t}}D_{i}\left(w_{i}(D)-\eta \right),$$

where for any given triplet (i,j,t) in ${𝒜}_{t},w_{i,j,t}(D)=P\left(H_{A,i,j,t}\cap H_{A,j,i,t}|Y\right)$ denotes the joint posterior probability that functional connectivity is detected between regions i and j from both BTV-PPI regressions, one treating region i as the target and the other with region j as the target. Since the two BTV-PPI regressions are fit independently, the joint posterior probability factors as $w_{i,j,t}(D)=P\left(H_{A,i,j,t}|Y\right)P\left(H_{A,j,i,t}|Y\right)$, which is then computed as

(13) $$\begin{array}{l} w_{i,j,t}(D)=P\left(\left|\beta_{i}^{j}(t)+\sum_{k=1}^{K}\;\gamma_{i,k}^{j}(t)S_{k}(t)\right|\geq c\sigma_{i}|Y\right)\\ \;\times P\left(\left|\beta_{j}^{i}(t)+\sum_{k=1}^{K}\;\gamma_{j,k}^{i}(t)S_{k}(t)\right|\geq c\sigma_{j}|Y\right), \end{array}$$

for some small c∈R. Our decision rule allows non-zero off-diagonal values in both $\Omega_{ij}(t)$ and $\Omega_{ji}(t)$ when the corresponding time-varying coefficients in (6) are both further than c standard deviations from 0 with high probability. In all the following implementations, we have found that c=0.1 suffices to obtain good operating characteristics.

Chandra and Bhattacharya (2019) suggest a multi-step approach to maximize the objective function in equation (12). First, they set the penalization constant $\eta =1-{mFDR}_{Y}$ where ${mFDR}_{Y}\in (0,1)$ indicates the *posterior modified false discovery rate* and is a measure of Type-I error in multiple hypothesis testing. This ${mFDR}_{Y}$ is defined as

(14) $${mFDR}_{Y}=\sum_{D\in 𝒟}\frac{\sum_{i\in {𝒜}_{t}}\;D_{i}\left(1-w_{i}(D)\right.}{\sum_{i\in {𝒜}_{t}}\;D_{i}}I(D|Y),$$

where 𝒟 denotes the space of all possible decision configurations and the indicator I(D∣Y) is equal to one *only* for the final decision.

Chandra and Bhattacharya (2019) use a simulated annealing method to maximize $f_{\eta ,t}(D)$ for a given η. However, the posterior of ${mFDR}_{Y}$ encodes an additional penalty for the incorrect decisions, particularly when the weights $w_{i,j,t}$ are small. Thus, using the ${mFDR}_{Y}$ to define the penalty may lead to an overly conservative procedure. As a way around the problem, they propose running the simulated annealing algorithm multiple times, progressively decreasing η until the desired ${mFDR}_{Y}$ is achieved. This approach is computationally burdensome for large datasets. Here, we employ an alternate approach, which has been motivated by the following realization. In our experiments, the achieved ${mFDR}_{Y}$ often falls below the nominal false discovery rate after the initial run of the simulated annealing algorithm, which leads to optimal decision pairs that are discordant, i.e. of the type $D_{i,j,t}\neq D_{j,i,t},i,j=1,\ldots ,P$. Subsequent runs of the annealing algorithm with progressively smaller η tend to reduce these discordant decisions. To improve computational efficiency, we introduce a simple modification after running the simulated annealing algorithm once. We inspect all the decisions and conclude that two regions are *not* associated only if the optimal solution identified by a single run of the annealing algorithm for a fixed η sets both $D_{i,j,t}=0$ and $D_{j,i,t}=0$. Thus we avoid an overly conservative decision procedure while only running the simulated annealing algorithm once. This modification is computationally efficient and appears to perform well in our investigations, striking a balance between accuracy and speed.

#### 2.3.2. Inference on Partial Correlations

Utilizing the non-marginal decision rules and our modified algorithm allows us to identify the structural zeros in our dynamic precision matrix, i.e. when inter-regional partial correlations are indistinguishable from 0. The last remaining step is to rectify those off-diagonal elements of Ω(t) that our decision process deemed statistically different from 0. Our approach is to take the average of the two off-diagonal entries, ensuring that Ω(t) is symmetric. We justify taking the average rather than some other function, such as the maximum, because we have no reason to prefer one estimate over the other after the selection algorithm has made a determination of significance. Our final fitted $\overset{ˆ}{\Omega }(t)$ has off-diagonal entries as follows:

$${\overset{ˆ}{\Omega }}_{i,j}(t)\;=\left\{\begin{array}{ll} 0 & \;\text{if}\;D_{i,j,t}=0\;\text{and}\;D_{j,i,t}=0\\ \frac{1}{2}\left({\tilde{\Omega }}_{i,j}(t)+{\tilde{\Omega }}_{j,i}(t)\right) & \;\text{otherwise}\; \end{array}\;i,j=1,\ldots ,R\right.$$

where, for any two regions i and j, the decisions $D_{i,j,t}$ and $D_{j,i,t}$ are the optimal decisions obtained by the simulated annealing algorithm of Chandra and Bhattacharya (2019) in the models fitted assuming target region i and j, respectively (see Section 3.2). Finally, from this fitted $\overset{ˆ}{\Omega }(t)$ that reflects our assumptions of sparsity in functional connectivity through our multi-comparison testing and also is symmetric, we can calculate inter-regional partial correlation according to equation (3).

Before transitioning to the applied portion of our paper, we briefly summarize the methodological framework. We are interested in obtaining potentially dynamic partial correlations between brain regions because they are directly interpretable, can control for drift in association that is independent of an experimental stimulus, and avoid problems with mediating effects. In Section 2.1, we discussed how partial correlations between brain regions can be derived from the precision matrix of a Gaussian Graphical Model, and that estimates of this precision matrix can be obtained from the generalized PPI model (gPPI). Building on this, in Section 2.2, we introduced the BTV-PPI model, which estimates the precision matrix while incorporating the desired time-varying dynamics, in contrast to the time-invariant nature of the gPPI model. Furthermore, in Section 2.3.1, we adapted a multi-comparison method for testing dependent hypotheses to identify structural zeros within the precision matrix. Finally, we obtained a final estimate of the precision matrix estimate over time by enforcing symmetry. This enables us to derive time-varying partial correlations between brain regions that vary through time and in response to a stimulus.

## 3. Results

### 3.1. Simulation Setup

In this section, we compare the estimates of partial correlation obtained using our proposed BTV-PPI model to those obtained using the standard gPPI model of McLaren et al. (2012). We show that our model can accurately detect an array of different partial correlation structures, including dynamic structures that the standard gPPI model can only loosely approximate.

We simulate fMRI data for P=15 different brain regions. We do this using a Gaussian Graphical Model with a specified precision matrix Ω(t) that varies over time. We choose the off-diagonal elements of the precision matrix to enforce certain properties of inter-regional partial correlation in simulated data. We have identified 5 different partial correlation structures we wish to have present in our simulated data in order to demonstrate that our proposed model is capable of recovering them all:

- 2 regions that are never functionally connected
- 2 regions that only have a PPI connection
- 2 regions that only have a constant, time-invariant physiological connection
- 2 regions that only have a dynamic physiological connection
- 2 regions that have both a physiological connection and a PPI effect

To construct Ω(t), we first decide which brain regions will share a physiological connection. We divide our 15 brain regions into 5 groups of 3 regions each. The regions within each group have either varying or time-invariant physiological connections. A time-invariant connection is achieved by setting $\Omega_{i,j}(t)=a,t=1,\ldots ,T$ for some scalar a with |a|<1 to help ensure invertability of Ω(t). A dynamic connection is achieved by setting $\Omega_{i,j}(t)=f(t)$ for some dynamic process f(t). Intra-group region pairs’ physiological connects are all set to 0, reflecting our assumption that most brain regions are not functionally connected. This is accomplished by setting $\Omega_{i,j}(t)=0,t=1,\ldots ,T$. The diagonal elements of Ω(t) are the inverse of the variance of our simulated data; we set these all equal to 1, so $\Omega_{i,i}(t)=1,i=1,\ldots ,P$. Finally, we then decide which region pairs’ partial correlations will have PPI effects. This is accomplished by adding an additional time course to the existing element of Ω(t). If the connection between regions i and j changes in the presence of the stimulus, then we add S(t)a for some scalar a to $\Omega_{i,j}(t)$. We tried two different version of the stimulus (convolved with the HRF). One which follows the traditional event-based experimental design where the underlying stimulus is a binary vector which indicates when the stimulus is “active.” We also tried another situation where the underlying stimulus is allowed to vary over time. We have not seen this situation in the literature but its exclusion seems unwarranted. There is no reason from a statistical perspective to preclude experimental setups where a subject is exposed to varying degrees of a stimulus. Indeed, our data application in the following section details just such a situation.

In this manner we construct two precision matrices, one for a binary stimulus and another for a variable stimulus, that both include all 5 of the desired partial correlation structures details above. We simulate 60 different datasets from each of these carefully constructed precision matrices. We then attempt to recover the true precision matrix by estimating its rows in a manner akin to Meinshausen and Bühlmann (2006)’s “neighborhood selection.” For comparison, we first estimate the rows with the standard gPPI model, where the coefficients do not vary over time. We then model the coefficients using penalized basis splines (Xue et al. 2020). Finally, we estimate the row with our proposed BTV-PPI model before and after the selection method of Chandra and Bhattacharya detailed in Section 2.3.1 as well as the subsequent correction for symmetry. We derive all inter-regional partial correlations from these four estimates of Ω(t) and compare them to the truth using the squared error from each simulation, defined as $SE=\sum_{t=1}^{T}\;\sum_{i=1}^{P}\;\sum_{j=1}^{P}\;{\left(\Omega_{i,j}(t)-{\overset{ˆ}{\Omega }}_{i,j}(t)\right)}^{2}$, and finding the mean over all 60 simulations.

### 3.2. Simulation Results

**Figure 2.**
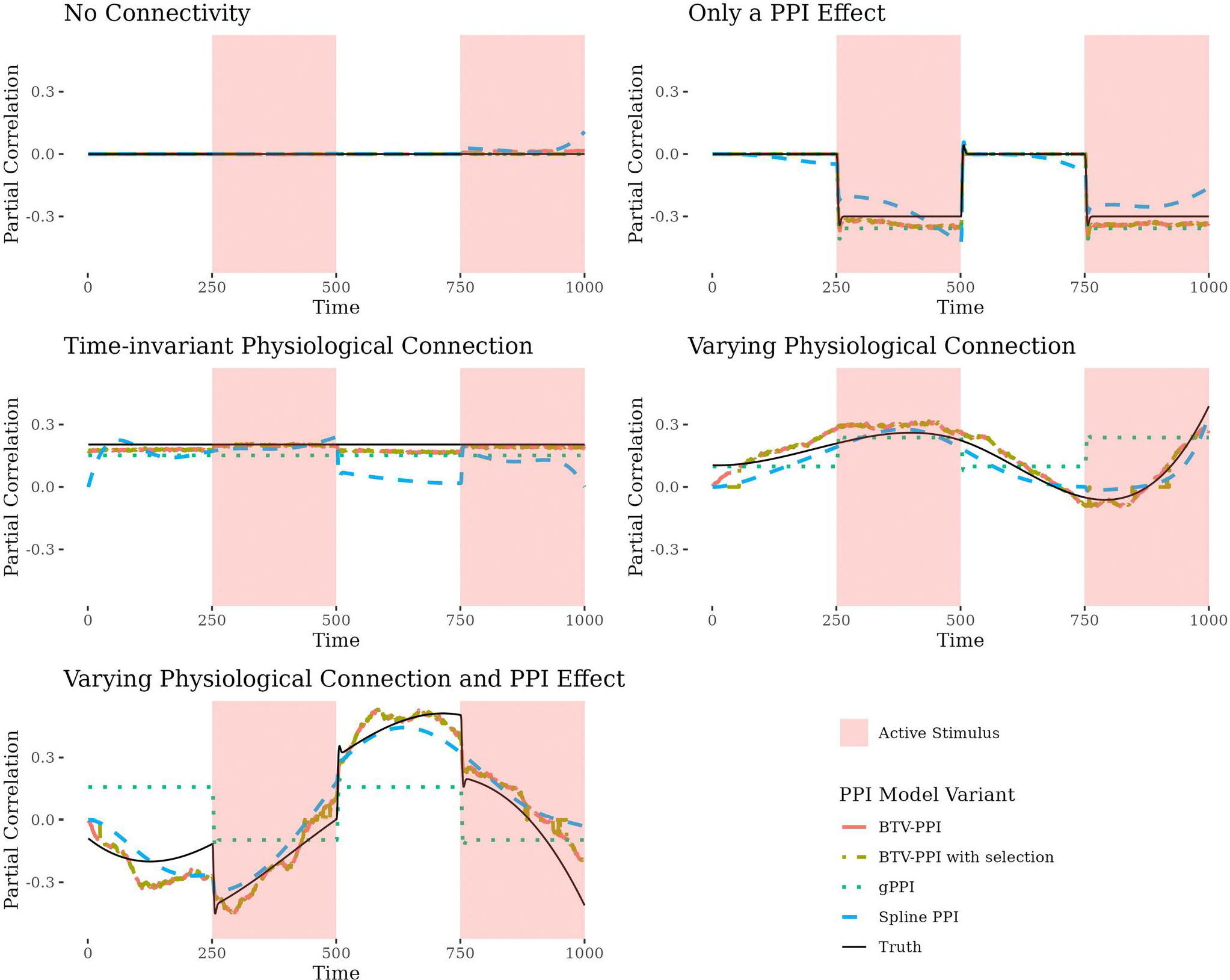
Simulation Study: estimates of partial correlation for 5 different scenarios. The estimates come from a single simulation with a *binary stimulus*. The plots compare the estimates arising from the generalized PPI (gPPI) model, the model fit using penalized basis splines (splinePPI), and our BTV-PPI model to the truth. Estimates are similar in scenarios where the physiological and PPI effects are time-invariant (the first three scenarios). The BTV-PPI model is better able to capture partial correlation when it varies over time independent of the stimulus (the last two scenarios).

We present a visual display of estimated partial correlation from a single simulation of data with a binary stimulus mimicking a block-design experiment in Figure 2. There are five scenarios we are interested in exploring, as mentioned above. The standard gPPI and our BTV-PPI models exhibit comparable performance in the first three scenarios, all of which lack time-varying partial correlations independent of the stimulus. This outcome is consistent with our expectation that the BTV-PPI model would perform well in scenarios where the gPPI model also performs well because our model generalizes the gPPI by allowing the coefficients to vary over time when necessary. In these scenarios, such flexibility is not required, leading the variance of the BTV-PPI coefficients to degenerate to zero, rendering them time-invariant. However, the BTV-PPI model outperforms the gPPI model in the final two scenarios, where physiological connectivity is allowed to vary. In these cases, the gPPI can only provide a loose approximation of the underlying partial correlations.

**Figure 3.**
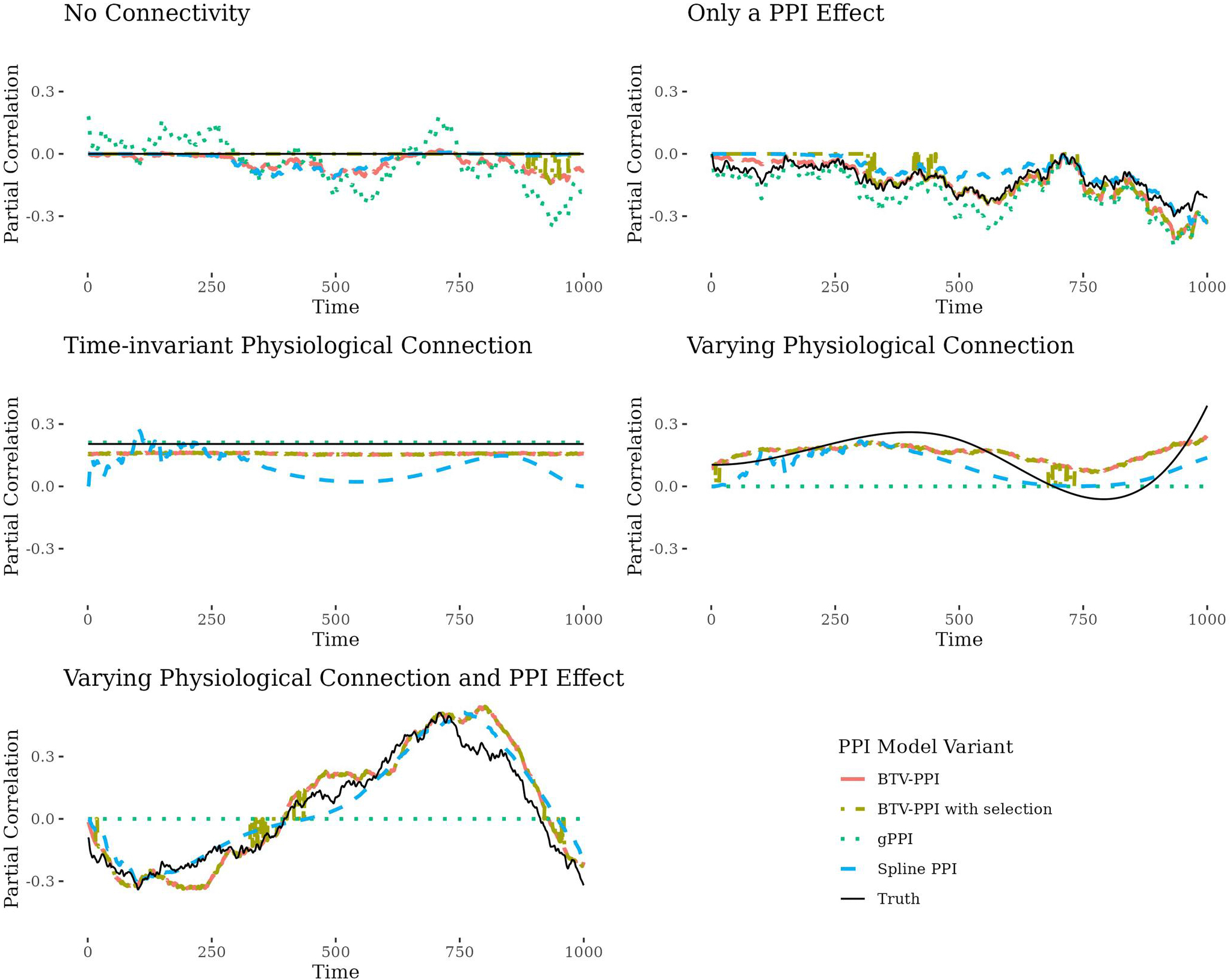
Simulation Study: estimates of partial correlation for 5 different scenarios. The estimates come from a single simulation with *varying stimulus strength*. The plots compare the estimates arising from the gPPI model, splinePPI model and our BTV-PPI model to the truth. The BTV-PPI model performs especially well when physiological connectivity varies and there is a PPI effect.

The ability of the BTV-PPI model to capture partial correlation in scenarios where physiological connectivity varies over time is more dramatic when the stimulus *strength* is also allowed to vary over time. A visual display of estimated partial correlation from a single simulation with varying stimulus strength, as described above, is presented in Figure 3. The last situation, which includes a PPI effect as well as varying physiological connectivity, demonstrates how inadequate the gPPI’s estimates of partial correlation can be.

We repeat these simulations 60 times, with the same Ω(t) corresponding to either a binary stimulus or variable stimulus, and calculate the mean squared error (MSE), defined as the average squared difference between each partial correlation estimate and the truth at each time point, for each of the 5 scenarios. The results for the binary stimulus are summarized in Table 1 and for the variable stimulus in Table 2.

### 3.3. Functional Connectivity Modulated by Lookahead Predictions

A topic of continuing interest in cognitive neuroscience is how individuals learn predictive associative relationships that can be used to support decision-making and planning for rewards (Daw and Shohamy 2008). Our interest lies in studying how learning in a predictive association experiment modulates functional connectivity. More specifically, we have available data on eight subjects who were shown a probabilistic sequence of four images. The subjects were asked to press one of four buttons that uniquely identified the image currently being shown (Bornstein and Daw 2012). The sequence of images was randomly generated (unknown to the participants) according to a first-order Markov process, where the probability a picture is shown in trial t=1,…,999 depends solely on the picture shown in trial t-1. The transition probabilities can be represented by a 4×4 matrix, where the (i,j) entry specifies the probability that picture j will be shown after picture i,i,j=1,…,4. Over the course of the experiment the subject implicitly learns these transition probabilities through experience. To encourage continual learning, the Markov transition matrix was changed twice, at trials t=334 and t=667. The participants’ reaction times (RTs), i.e. how quickly a subject took to correctly identify the current image, were measured as a proxy for predictive learning; a faster reaction time indicates that a subject relied to some degree on a prediction of the picture they were subsequently shown. The true transition matrix, which the subject was implicitly learning, was unique to each subject and selected to minimize mixing time, the time it takes for a Markov Process to be close to its steady state. This was done so that first-order dependencies would be the only consistent source of information about the next image and could be used as the primary predictor of behavior. In the following section we detail how we derive regressors that represent learning under different, empirically justified assumptions to include in our BTV-PPI model.

#### 3.3.1. Learning Rules and Estimation of the Lookahead Activity

The relationship between the RTs and the underlying stimulus probabilities is typically captured by the learning rate, which measures the weight a system places on new information relative to previous experience. Following previous work on the division of learning into multiple systems (Gläscher and Büchel 2005; Poldrack et al. 2001), we consider two Rescorla-Wagner learning rules under an assumption of either a slow or a fast learning rate: $P^{s},P^{f}\in {ℛ}^{4\times 4\times 999}$ where $P^{s}$ and $P^{f}$ represent the evolution of a subject’s learned transition matrix probabilities over time according to a slow learning rate and a fast learning rate respectively. The evolution of the learned transition matrix is described by

(15) $$P_{i,j,t}^{z}=\;\left\{\begin{array}{ll} P_{i,j,t-1}^{z}+\alpha_{z}\left(1-P_{i,j,t-1}^{z}\right) & j=I(t)\\ P_{i,j,t-1}^{z}+\alpha_{z}\left(0-P_{i,j,t-1}^{z}\right) & j\neq I(t) \end{array}\;z=s,f;\;t=1,\ldots ,999\right.$$

where I(t)∈1,2,3,4 indicates the label of the image shown at trial t=1,…,999. This learning rule models how a subject updates their transition probabilities with each image presented. $P_{i,j,t-1}^{z}$ is a subject’s learned transition matrix before the image in trial t is shown, i.e. their experience up until t, and $\alpha_{z}$ is the weight given to new information received in trial t, the learning rate. We use $\alpha_{s}=0.0138$ and $\alpha_{f}=0.5499$, the median parameter values across the broader population, as estimated in Bornstein and Daw (2012). We also assume the subjects place equal weight on all transitions at the start of the experiment, i.e. $P_{i,j,0}^{z}=1/4$. Using this starting value, the two assumed learning rates and the observed image sequence we compute $P^{s}$ and $P^{f}$ and from these derive the *forward entropy* of the system under the two learning rates, say $H^{s}(t)$ and $H^{f}(t)$ for the slow and fast rates respectively. The forward entropy is a measure of the expected surprise of the next image given the participant’s current experience and the image they are currently viewing under each assumed learning rate. It captures the amount of *lookahead* activity to be expected in anticipation of the upcoming stimulus (Bornstein and Daw 2013; Johnson and Redish 2007; Khoudary et al. 2022; Wang et al. 2022):

(16) $$H^{z}(t+1)\;=E\left[-log\left(P_{I(t),j,t}^{z}\right)\right]\;\;=-\sum_{i=1}^{4}\;log\left(P_{i,j,t}^{z}\right)P_{i,j,t}^{z}\;z=s,f$$

**Figure 4.**
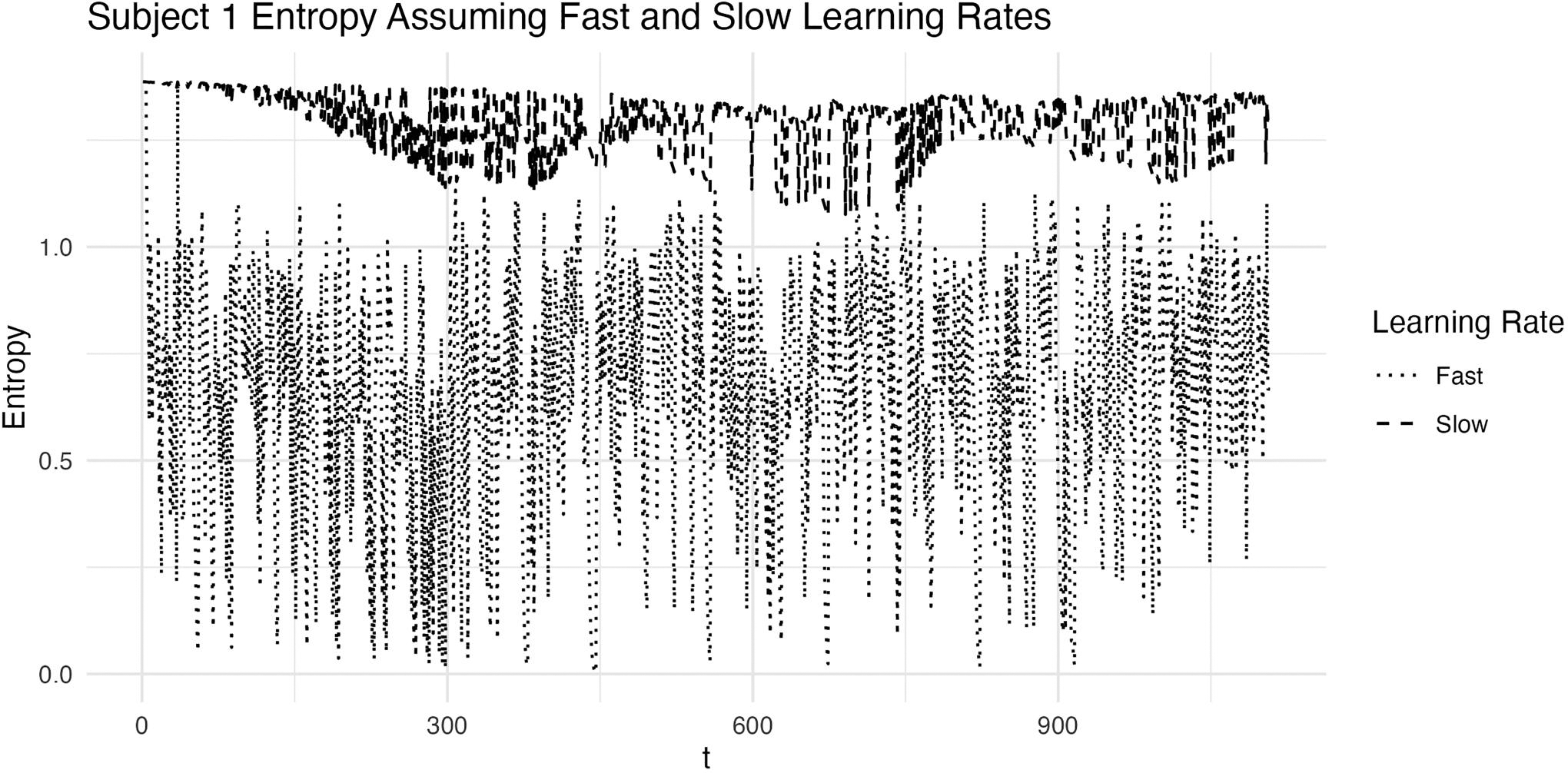
An example of lookahead entropy under the two assumed learning rates for a subject in the reaction time experiment of Section 3.3.

Figure 4 gives an example of the calculated lookahead entropies under the two assumed learning rates given the actual sequence of images presented during the experiment. Both lines start at the same value at t=1, which reflects an expectation that the subject places equal weight on each of the four images at the beginning of the experiment. Lookahead entropy under a fast learning rate, represented by the dotted line, quickly drops to a lower value while forward entropy under a slow learning rate exhibits much smaller sequential jumps. This illustrates how a fast learning rate yields predictions with more confidence, and thus lower entropy, because more weight is given to very recent experience and thus there is less ambiguity in the predicted next image. Predictions under a slow learning rate, by contrast, come with less certainty, reflecting conflicting experiences from a broader window of time. The original study found evidence that different brain regions were predictive of RTs under different assumed learning rates. In particular, the Hippocampal region was predictive of RTs under the slow learning rate while the Striatum was predictive of RTs under the fast learning rate. These results motivate our investigation into whether functional connectivity changes in response to the entropy under the two learning rates. We also use the data from this study to illustrate other interesting patterns of functional connectivity that our proposed BTV-PPI model is able to uncover.

#### 3.3.2. Time-Varying Psychophysiological Interaction and Lookahead Activity

In this section, we fit our model to the data described in the previous section and explore some different patterns of functional connectivity that we uncover. As in the simulated data setting, our proposed BTV-PPI model allows us to identify several classes of relationships between brain regions that the traditional PPI model cannot. The types of relationships the model can identify include pairs of regions that are functionally connected with either constant or varying strength that is independent of the experimental conditions as well as pairs of regions whose functional connectivity varies in response to experimental conditions.

Imaging was performed on the 3T Siemens Allegra head only scanner with time resolution of 2.0s per acquisition, across four sessions of 300 acquisitions each. Images were normalized into a template and resampled into 2 × 2 × 2-mm voxels in the normalized template space (MNI). The voxels were then further combined into P=18 regions of interest (ROIs) by taking the mean BOLD signal of all voxel time series attributed to each ROI. The ROIs were defined anatomically according to the standard atlas (AAL). The regions selected were structures previously associated with learning, memory, and decision-making, along with several “control” regions, and separated bilaterally. We consider K=4 stimulus covariates of interest in the model:

- An indicator for the presentation of an image
- An indicator for the subject pressing a button
- The forward entropy under the slow learning rate: $H^{s}(\cdot )$
- The forward entropy under the fast learning rate: $H^{f}(\cdot )$

Each stimulus covariate was convolved with the canonical HRF using the finite impulse response method, detailed in Section 2.1, to create the psychological regressors. PPI regressors were formed from the convolved product of each stimulus and each region’s BOLD response.

We fit our model to the data described above. The additive nature of the partial correlations in our model makes it possible to separate the contributions of physiologicial (background) connectivity and PPI effects before the selection of non-zero components described in Section 2.3.1 is performed. We show some examples of patterns of partial correlation for a single subject to illustrate the types of relationships the BTV-PPI model is able to capture.

### 3.4. Applied Results

**Figure 5.**
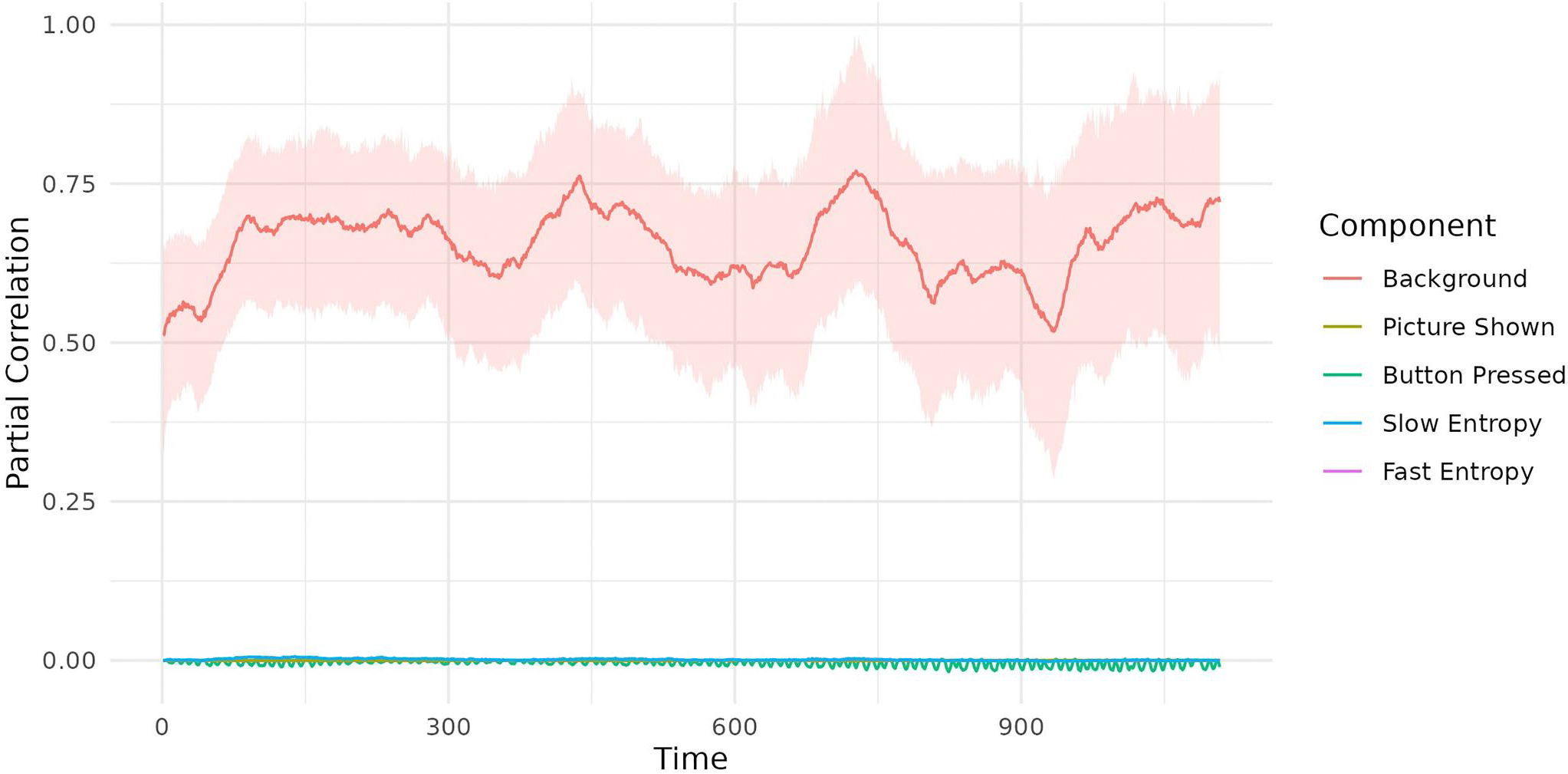
RT experiment: Partial Correlation between Left and Right Anterior Cingulate Cortex for a single representative subject. The physiological (background) connectivity varies over time, independent of the experimental conditions. We have included the 90% Credible Interval for only the Background functional connectivity component. See Section 3.4 for details.

**Figure 6.**
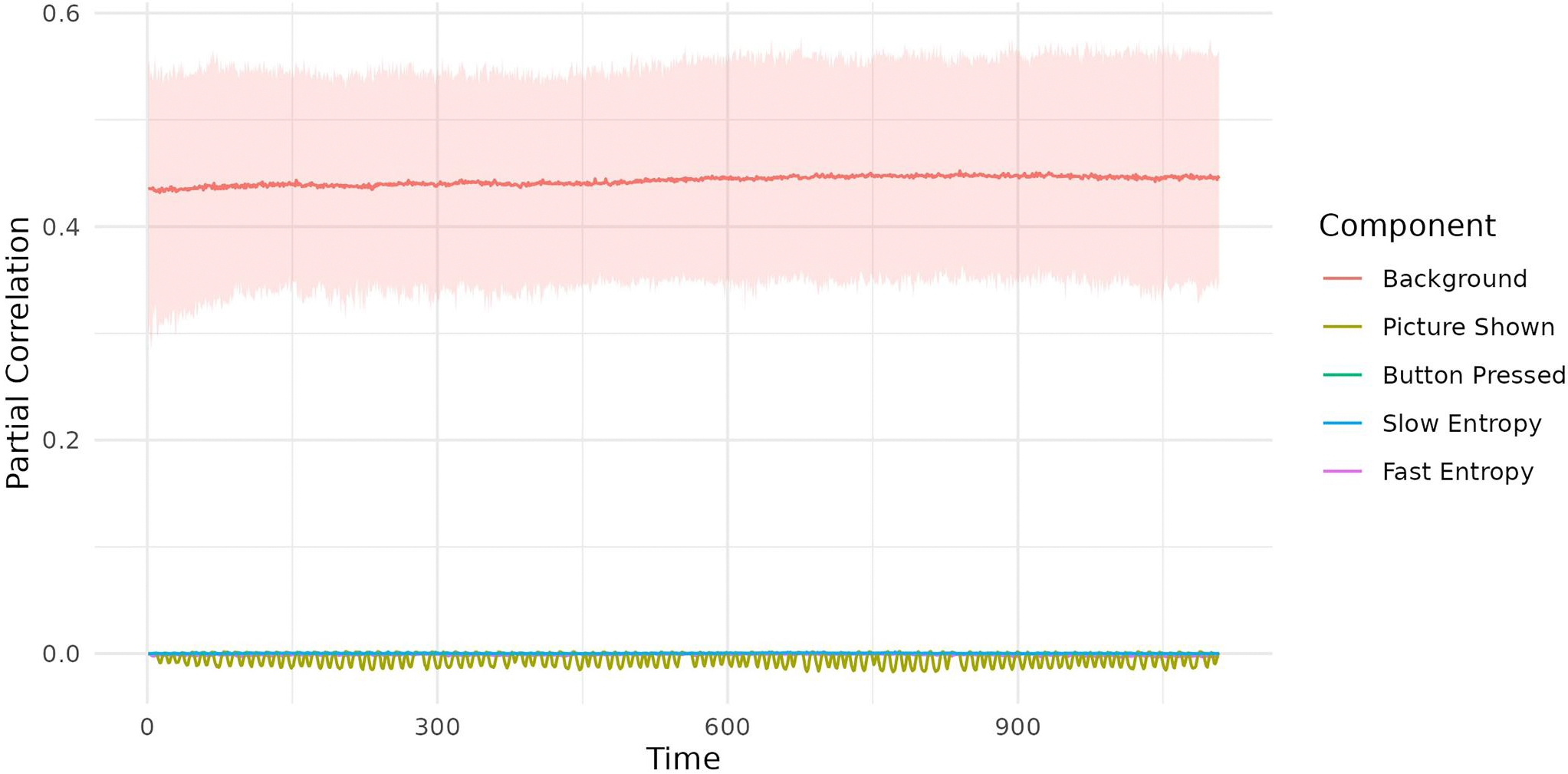
RT experiment: Partial Correlation between the Left and Right Hippocampus for a single representative subject. The physiological connectivity remains constant over the course of the experiment. We have included the 90% Credible Interval for only the Background functional connectivity component. See Section 3.4 for details.

We observe that the corresponding left and right sides of most brain structures across all subjects exhibit a strong degree of functional connectivity. For some brain regions this connectivity varies in strength independent of the experimental conditions. For instance, the left and right sides of the Anterior Cingulate Cortex region exhibit this pattern (Figure 5). By contrast, the left and right sides of the Hippocampus do not show this fluctuating pattern; the partial correlation between these two regions remains fixed over the course of the experiment for this subject (Figure 6).

**Figure 7.**
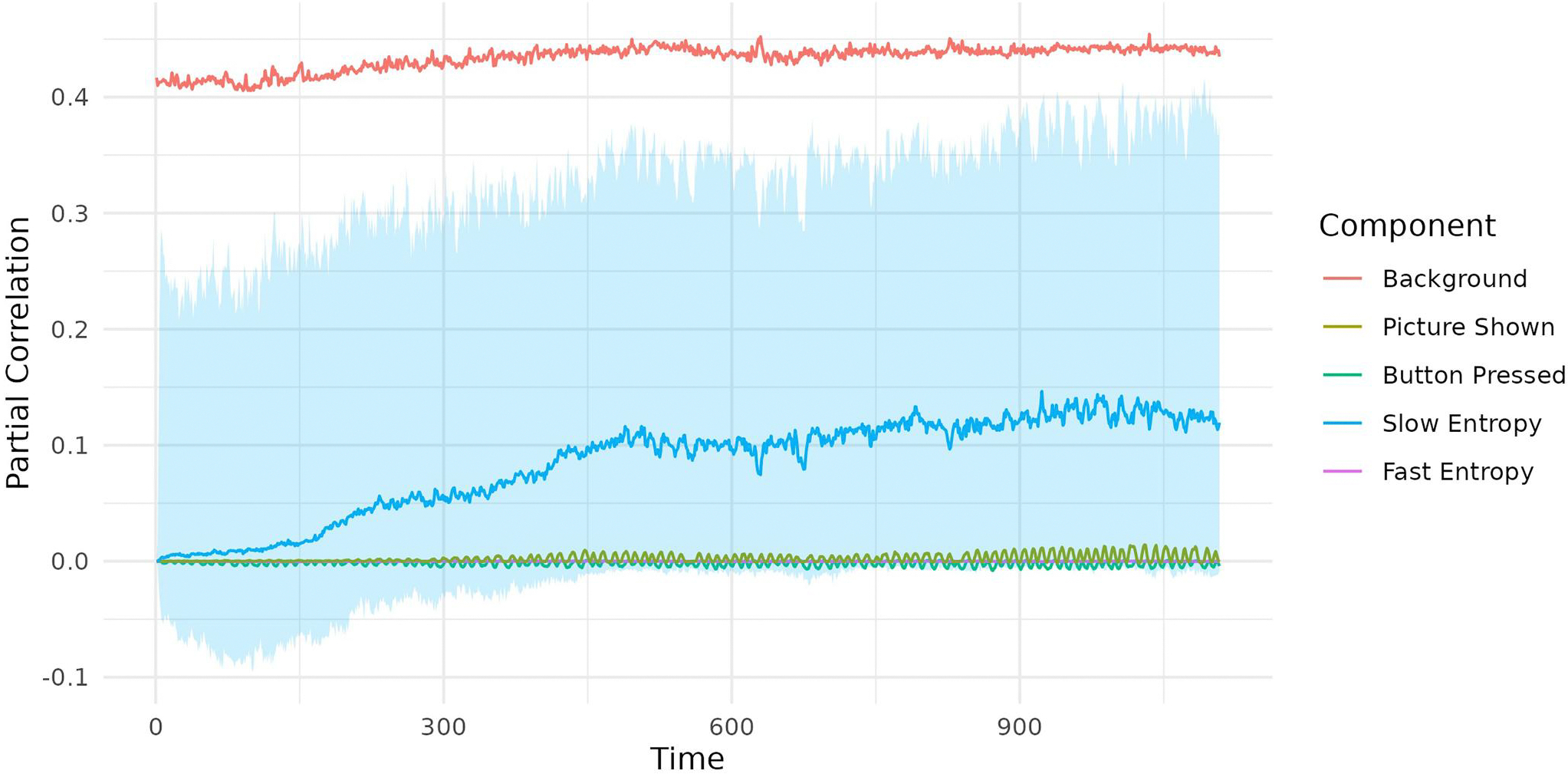
RT experiment: partial correlation between the left and right Caudate for a single representative subject. While physiological (background) connectivity remains constant, a PPI effect from slow entropy gradually increases over the course of the experiment. We have included the 90% Credible Interval for only the functional connectivity component associated with slow entropy. See Section 3.4 for details.

We also see pairs of brain regions with functional connectivity that is modulated by the experimental conditions. For example, the total partial correlation between the left and right sides of the Caudate region for that same subject increases over the course of the experiment (Figure 7). This increase is not due to a change in the physiological (background) connectivity, which remains constant, but to the PPI effect of slow forward entropy. It’s not clear why the coordination of activity in the two sides of the Caudate region would increase in response to slow entropy; one possibility is that the region is being recruited for predictions when the alternative predictive system is uncertain, which would be consistent with extensive theoretical work on uncertainty-weighted arbitration in multiple learning systems (Daw et al. 2005; Keramati et al. 2011; Lengyel and Dayan 2007; Wang et al. 2022). This result could motivate future research into the role this region plays in predictive tasks where the reliability of learning changes differentially across a task.

**Figure 8.**
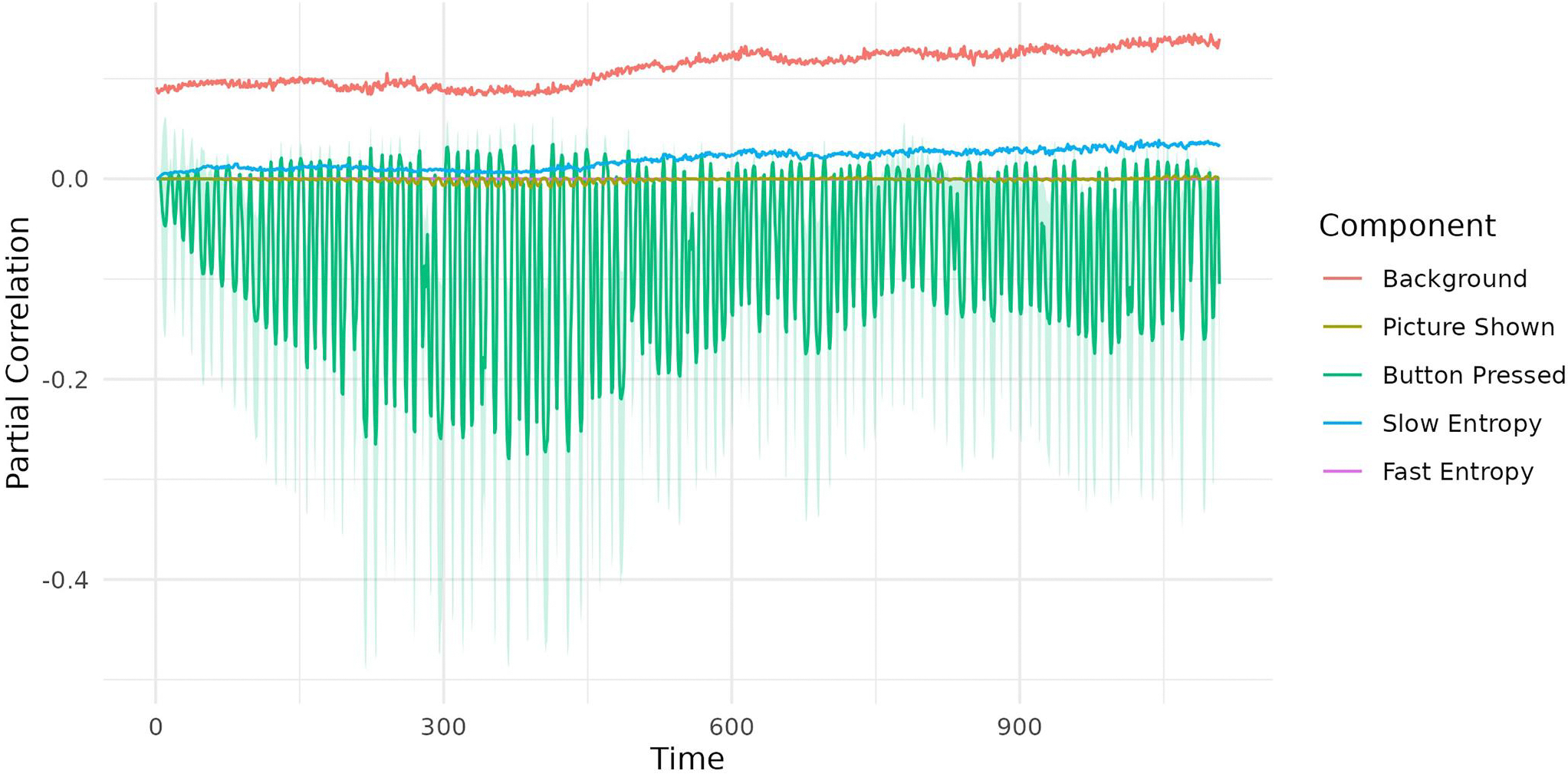
RT experiment: partial correlation between left Hippocampus and left Nucleus Accumbens for a single representative subject. A strong PPI effect is associated with a button being pressed. We have included the 90% Credible Interval for only the functional connectivity component associated with the press of a button. See Section 3.4 for details.

As a further example of functional connectivity that changes in response to experimental conditions, consider the left Hippocampus and left Nucleus Accumbens (Figure 8), which exhibit a pronounced PPI effect corresponding to the press of a button. The direction of this effect is also interesting. While the physiological connectivity is constant over the course of the experiment, the PPI effect of a button press negates that coordination or even causes the activity in the two regions to be anticorrelated, consistent with the quenching of neural variability at response execution typically observed in decision-making tasks (Churchland et al. 2010). This observation is further consistent with prior work suggesting that these regions have a coordinated role in motor control (Poldrack et al. 2001).

**Figure 9.**
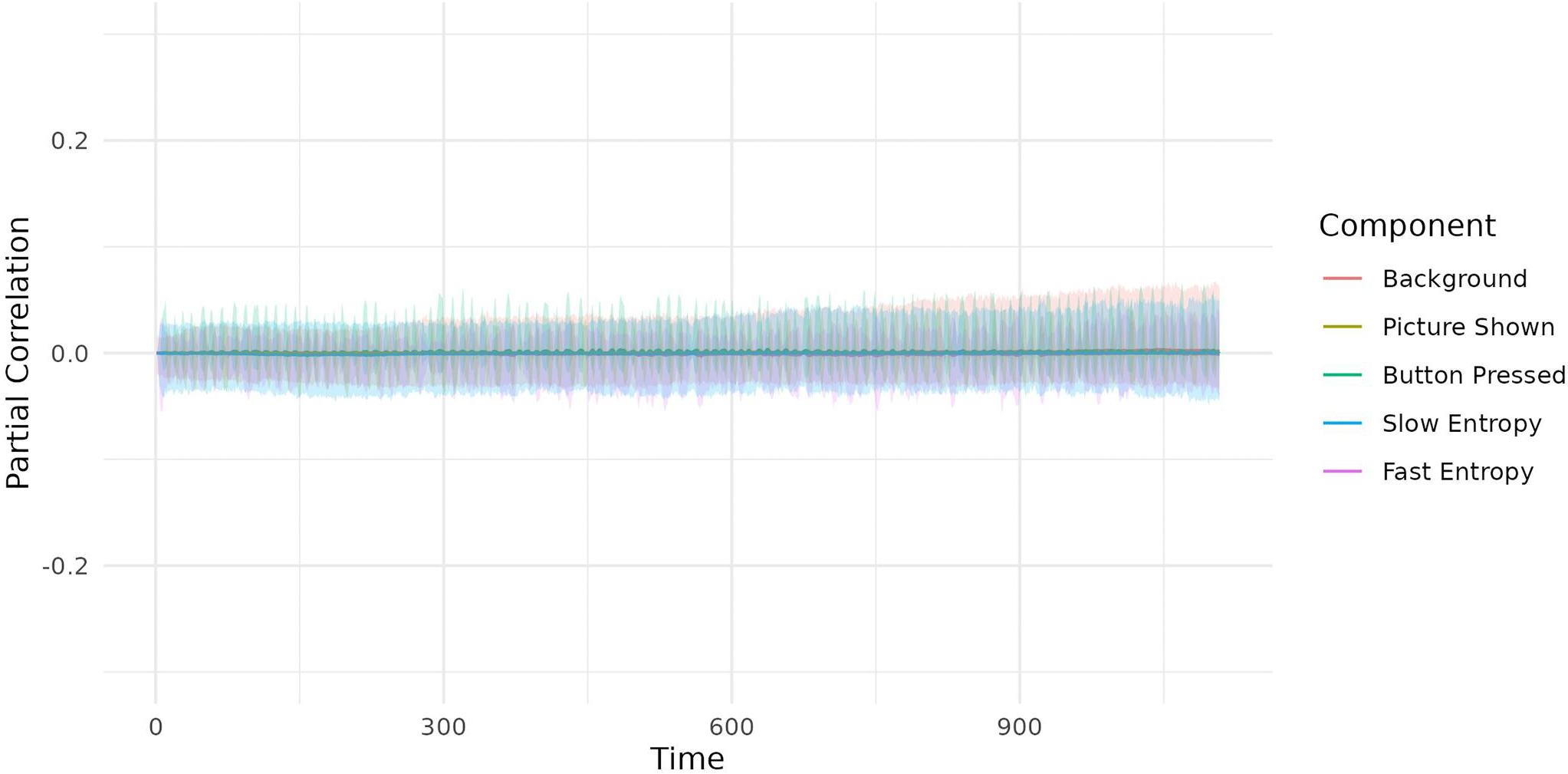
RT experiment: partial correlation between left Anterior Cingulate Cortex andleft Caudate for a single representative subject. There is no functional connec-tivity over the course of the experiment. We have included the 90% CredibleInterval for all functional connectivity components to emphasize that they areall tightly centered around 0. See Section 3.4 for details.

Finally, we note that many pairs of brain regions under consideration had no functional connectivity over the course of the experiment, even before the non-zero component selection process of Section 2.3.1. This aligns with an expectation of sparsity among the functional connections within the brain. As an example, we include a graph of the components of partial correlation between the left Anterior Cingulate Cortex and the left Caudate (Figure 9).

In Section 3.2, the simulation results, we compared the performance of different PPI model configurations by looking at how well each of them estimated the known partial correlations. We did this by calculating the mean squared error between the estimated partial correlation and the truth. In this applied analysis we do not know the true partial correlations so cannot calculate the MSE. To demonstrate the advantage of using the BTV-PPI over the standard gPPI in this setting, we argue that the BTV-PPI model better explains each seed region’s BOLD activity than the gPPI so the partial correlations derived from the BTV-PPI’s estimated time-varying coefficients must also be better. The MSE between the observed BOLD response in region $i,\;Y_{i}(t)$, and the estimated BOLD response ${\overset{ˆ}{Y}}_{i}(t)$ is 0.29 when the BTV-PPI model is used but 0.39 when the gPPI model is used. The proportion of the variation in BOLD activity explained by each model can also be used for comparison. This value is known as $R^{2}$. When the BTV-PPI is used $R^{2}=0.70$ but when the gPPI is used $R^{2}=0.61$ (see Supplemental Materials for more details). This shows that the BTV-PPI model better fits the data and we argue that it consequently yields better estimates of partial correlation.

## 4. Discussion

We have introduced a novel Bayesian model able to estimate potentially dynamic partial correlations arising both from drift in background functional connectivity and in response to experimental stimuli. The method involves fitting P independent linear regressions with time-varying coefficients and then using a non-marginal decision framework that identifies structural zeros in the precision matrix of a Gaussian Graphical Model. This framework also enforces the assumption of the undirected nature of functional connectivity by ensuring the symmetry of the precision matrix.

In simulations, our proposed BTV-PPI model performs comparably to the standard gPPI model in scenarios where partial correlations remain time-invariant but significantly outperforms the gPPI in scenarios where partial correlations vary over time. Importantly, we demonstrated how the gPPI model may erroneously infer a psychophysiological interaction between regions when, in fact, only a varying physiological connection is present. This highlights a critical limitation the gPPI model and underscores the need for approaches that can accurately account for such variability.

Additionally, we showed that the BTV-PPI model can accommodate continuous experimental predictors, expanding its applicability beyond block or event-related stimuli. We believe that this feature addresses a gap in current modeling frameworks and hope that future research explores the possibility of time-varying continuous experimental conditions.

In an application of our model to a predictive learning experiment, we established the presence of distinct patterns of functional connectivity. Focusing on a single subject, we identified several unique patterns in the partial correlation between different brain region pairs. These patterns may be consistent with the dynamic arbitration between multiple decision and learning systems that has been proposed in the literature (Daw et al. 2005; Wang et al. 2022) and observed in behavior (Yoo and Bornstein 2024) but rarely observed in neural activity, perhaps due to insufficiently sensitive statistical tools. Notably, the patterns exhibiting dynamic partial correlation in either the physiological or PPI components could not be captured by the gPPI model.

A key innovation of our model is its ability to estimate a precision matrix that describes multivariate data varying both in response to experimental conditions and incrementally, independent of those conditions. Furthermore, the model is able to disambiguate between those two sources of variability.

However a limitation of the proposed framework is that this model can be fully applied only to single subject’s data. Attempts to find patterns of partial correlation that are consistent across all subjects in a study may face several challenges.

First, allowing each subject’s physiological connectivity to drift over time makes direct comparisons of partial correlation time courses across subjects difficult. Although the magnitudes of partial correlations may be generally comparable for specific brain region pairs, inter-subject differences may not be considered meaningful. Additionally, there may be significant heterogeneity in the PPI responses to different stimuli across subjects. For example,in the reaction time experiment considered in Section 3.3, while one subject exhibited a strong change in partial correlation between the Hippocampus and Nucleus Accumbens associated with button presses, other subjects displayed a weaker or absent effect.

Consequently, we emphasize that the results of our applied analysis are not intended to suggest universal patterns of association but rather to demonstrate the model’s capacity to uncover distinct patterns where they exist. An important avenue for future research is the extension of these types of model-based frameworks for studying time-varying connectivity in complex experiments from subject-level models to group-level analyses.

## Supplementary Material

Supplementary Materials

Supplemental data for this article can be accessed online at https://doi.org/10.1080/26941899.2025.2519436.

## Figures and Tables

**Table 1.** Mean squared error (and standard deviation) across 60 simulations with a binary experimental stimulus. The minimum MSE for each class of connectivity are emphasized in **bold**.

Results with a Binary Experimental Stimulus				

	PPI model variant			
Connectivity type	gPPI	spline-PPI	BTV-PPI	BTV-PPI with selection

No Connectivity	0.82(2.54)	0.34(0.53)	0.33(0.84)	**0.06**(0.5)
Only a PPI Effect	184.93(36.17)	10.93(5.25)	2.11(2.01)	**2.09**(2.04)
Time-invariant Physiological Connection	167.74(31.12)	9.68(3.91)	**1.48**(2.04)	2.01(3.3)
Varying Physiological Connection	65.75(26.12)	9.4(4.94)	**8.26**(3.9)	10.65(6.02)
Varying Physiological Connection and PPI Effect	137.52(16.59)	14.3(4.89)	**7.74**(3.41)	9.24(3.74)

**Table 2.** Mean squared error (and standard deviation) across 60 simulations with a time-varying continuous experimental stimulus. The minimum MSE for each class of connectivity are emphasized in **bold**.

Results with a time-varying continuous experimental stimulus				

	PPI model variant			
Connectivity type	gPPI	spline-PPI	BTV-PPI	BTV-PPI with selection

No Connectivity	1.97(5.69)	0.19(0.35)	0.18(0.48)	**0.01**(0.06)
Only a PPI Effect	75.32(51.01)	8.77(3.63)	**2.07**(1.56)	3.62(3.03)
Time-invariant Physiological Connection	157(55.53)	9.63(3.7)	**1.82**(2.04)	2.37(2.98)
Varying Physiological Connection	60.19(33.96)	8.61(3.5)	**6.73**(2.64)	9.25(5.22)
Varying Physiological Connection and PPI Effect	65.2(8.1)	11.11(3.48)	**7.48**(3.48)	9.22(3.95)
