# Supplementary material for: A Bayesian Time-Varying Psychophysiological Interaction Model
Source: Data Sci Sci. Author manuscript; Available in PMC 2026 Jun 11. (PMC13251719; doi:10.1080/26941899.2025.2519436)
Supplement: Supplementary Materials [file NIHMS2172314-supplement-Supplementary_Materials.pdf]

Supplemental Materials

1. Brain ROIs Included in Applied Analysis

In the applied analysis portion of this article we included 18 brain regions of interest (ROIs). These were the left and right (bilateral) structures of:

(1) Anterior Cingulate Cortex

(2) Caudate

(3) Fusiform Gyrus

(4) Hippocampus

(5) Lateral Occipital Cortex

(6) Lingual Gyrus

(7) Nucleus Accumbens

(8) Parahippocampal Cortex

(9) Putamen

These regions were selected because they were significantly active in response to the regressors of interest in the original study (Bornstein and Daw 2012) (Hippocampus, Caudate, Nucleus Accumbens, Putamen and Anterior Cingulate Cortex), or because they are widely associated with responses to the content category of the images used as stimuli (Lateral Occipital Cortex, Fusiform Gyrus, Lingual Gyrus, Parahippocampal Cortex).

2. Improved Fit of BTV-PPI Model over the gPPI Model

Below we plot the Mean Squared Error and R^2 for each of the 18 seed regions using both the gPPI and the BTV-PPI models. We connect each region's MSE and R^2 using the two models with a line so that it is visually apparent that the fit for each region improves when using the BTV-PPI over the gPPI. For the MSE plot, this is clear in the downward-sloping lines. The regions that do not show much of an improvement when the BTV-PPI is used are those that do not have time-varying functional connectivity with other regions. Those regions that do show a dramatic decrease in MSE when the BTV-PPI is used.

The figure for R^2 tells a similar story. R^2 is a measure of the proportion of variation in the data from the seed region that can be explained by the model. The upward sloping lines show that the BTV-PPI model is able to explain more of the variation in all regions compared to the gPPI.

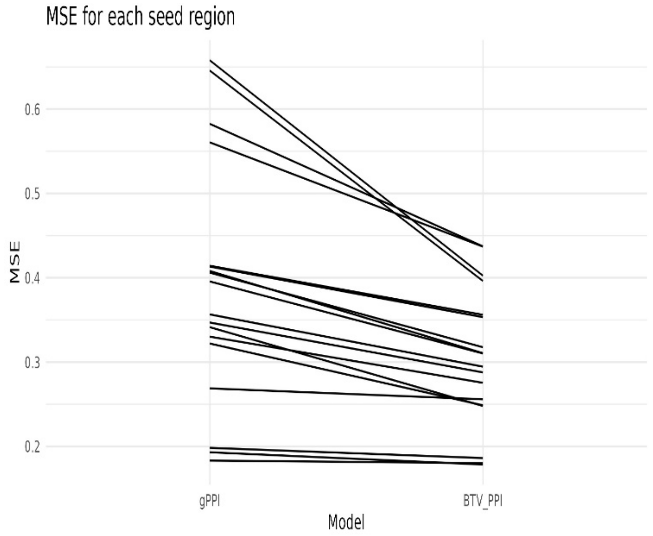

Figure 10. The Mean Squared Error for each of the 18 seed regions when the gPPI and BTV-PPI model are used. A line connects region's two measurements to show that while there is overlap in the overall distribution of MSE values, each individual region has a smaller MSE when the BTV-PPI model is used.

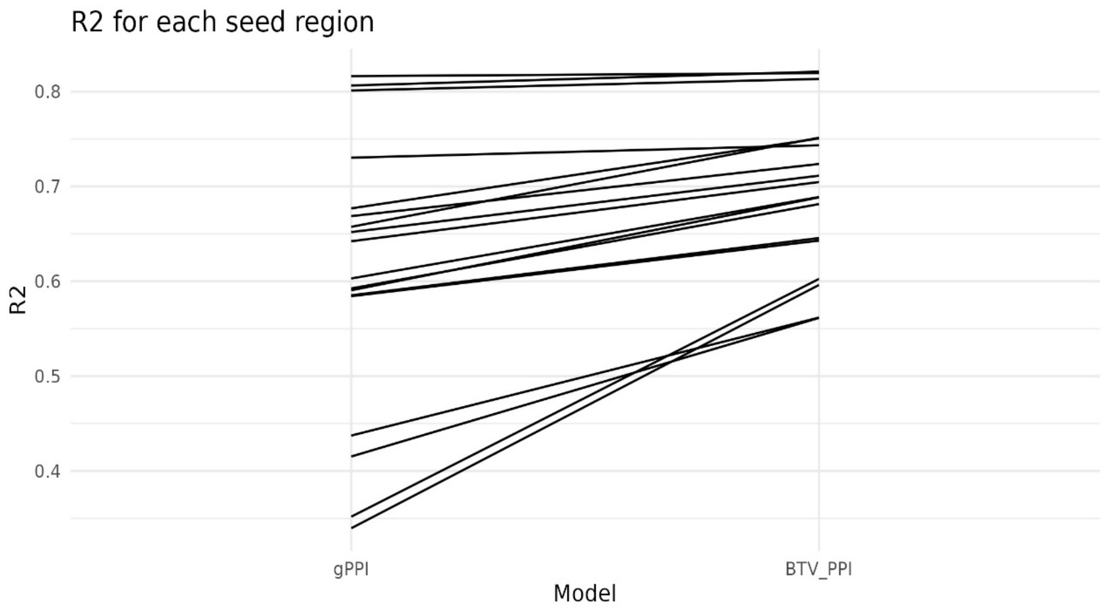

Figure 11. The proportion of variation in the data from each seed region explained by the gPPI and BTVPPI models. The upward sloping lines for all regions indicate that the BTV-PPI model fits each region's data better than the gPPI.
